# One-pot CRISPR-based point of care platform for rapid, specific and sensitive detection of HPV 16 without pre-amplification

**DOI:** 10.1038/s41378-025-01130-y

**Published:** 2026-03-09

**Authors:** Yalin Chen, Yicheng Chen, Cuijuan Zhang, Yongsheng Cai, Zhuoer Zeng, Julien Reboud, Jonathan M. Cooper, Hongbo Shan, Yang Wang, Gaolian Xu

**Affiliations:** 1 School of Biomedical Engineering, Med-X Research Institute, Shanghai Jiao Tong University, Shanghai, China; 2 Key Laboratory of Biomechanics and Mechanobiology, Ministry of Education, Beijing Advanced Innovation Center for Biomedical Engineering, School of Engineering Medicine, Beihang University, Beijing, China; 3 Department of Cardiac vascular surgery, the First Medical Center, Chinese PLA General Hospital, Beijing, China; 4 Department of Thoracic Surgery, Beijing Tiantan Hospital, Capital Medical University, Beijing, China; 5 Division of Biomedical Engineering, University of Glasgow, Glasgow, UK; 6 Adicon Clinical Laboratories, Hangzhou, Zhejiang China; 7 Shanghai Sci-Tech InnoCenter for Infection and Immunity, Shanghai, China; 8 The Institute of Infection and Health Research, Fudan University, Shanghai, China

**Keywords:** Electrical and electronic engineering, Biosensors

## Abstract

Accurate detection of gene subtypes with high sequence similarity is critical for pathogen diagnosis. Current CRISPR-based PCR diagnostics methods may provide improved specificity but rely on pre-amplification in a separate reaction, due to Cas protein thermal instability, increasing cross contamination. Here, we developed CRISPR-based terminal-specific amplification (CASTSA), a one-pot platform which makes use of the CRISPR-Cas12a specific recognition and cleavage, generating a single strand digested product with specific 5’ termini, to serve as the template for qPCR amplification. Our assay simplifies sample preparation by eliminating the need pre-amplification, whilst simultaneously fully exploiting the high specificity of the CRISPR system and high sensitivity of PCR. CASTSA was validated in vitro and with clinical samples collected from individuals with Human Papillomavirus (HPV), demonstrating high specificity for HPV 16, whilst discriminating HPV 18, 33, 45, and 52 sub-types, using a laser-induced graphene (LIG)-based electrochemical sensor platform. The technique achieved a limit of detection of 18 copies/reaction and offers a robust and reproducible, one-pot solution for pathogen subtyping, providing excellent specificity, so advancing nucleic acid detection with an assay that is easier to implement when compared with standard clinical diagnostic workflows.

The rapid and accurate detection of nucleic acid biomarkers, particularly those with high sequence similarity, is essential for pathogen diagnosis and treatment.^1^ Traditional real-time quantitative polymerase chain reaction (qPCR) faces significant limitations in subtyping applications.^2^ In these situations, primer and probe binding regions closely resemble non-target sequences,^3^ leading to competitive hybridization, compromising the specific amplification of target sequences and increasing the risk of false positives.

Clustered regularly interspaced short palindromic repeats (CRISPR), in combination with CRISPR-associated (Cas) proteins, have been widely explored to enhance the specificity of nucleic acid detection.^4^ Guided by CRISPR RNA (crRNA), the Cas protein-crRNA complex identifies and cleaves target sequences at specific sites through *cis*-cleavage activity, enabling precise recognition and greater specificity compared to conventional primer-based approaches.^5–7^ CRISPR-based diagnostics (CRISPR-Dx) have been successfully applied to the detection of various pathogens,^8,9^ mutations,^10,11^ and single-nucleotide polymorphisms (SNPs).^12^ Prominent CRISPR-Dx assays such as SHERLOCK,^13^ DETECTR,^14^ and HOLMES,^15^ typically involve pre-amplification of target sequences using PCR^16^ or isothermal methods (e.g., LAMP,^17^ RPA,^18,19^ RCA^20,21^), followed by CRISPR-mediated *trans*-cleavage detection. The reliance on pre-amplification limits specificity, as nonspecific amplicons generated during this step cannot be fully excluded by crRNA’s secondary recognition.^22^ Additionally, the PCR pre-amplification is incompatible with carrying the reaction in a single tube (“one-tube” or “one-pot”), increasing the risk of cross-contamination during intermediate transfer steps.^15,23^

In this work, we developed CRISPR-based terminal-specific amplification (CASTSA), a one-pot nucleic acid detection platform that enhances specificity while avoiding cross-contamination. CASTSA employs the Cas12a ribonucleoprotein (RNP) effector to directly recognize and cleave target sequences, generating single-stranded 5’ terminal that initiate qPCR amplification, while leaving the undigested sequence without amplification (Fig. 1). This approach fully exploits the high specificity of the CRISPR system while overcoming Cas protein denaturation during PCR, and ensures strict control over the amplification process. The platform utilizes the LbaCas12a system, which offers better duplex stability and lower mismatch tolerance compared to Cas9, due to its shorter single-strand crRNA and the formation of an intramolecular R-loop structure.^24^ A terminal-specific primer (TSP) was designed to recognize the digested product of CRISPR/Cas and initiate qPCR, by forming a functional self-folding structure that reduces nonspecific primer binding.

**Fig. 1:**
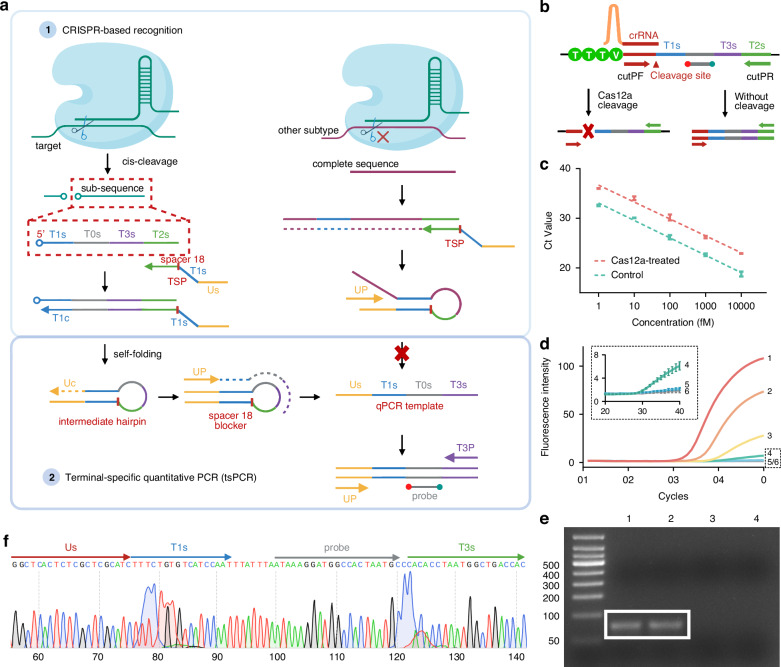
CASTSA mechanism. **a** Detection when recognizing the target nucleic acid (left) and with other non-specific subtypes with partial similar regions (right). **b** Design of primers cutPF and cutPR for LbaCas12a *cis-*cleavage validation. **c** Comparison of the amplification efficiency when using cutPF/PR for targets treated with Cas12a and a non-treated control for *cis-*cleavage validation. **d** Feasibility of tsPCR using the single-strand template. (1) 100 fM, (2) 10 fM, (3) 1 fM, (4) 100 aM, (5) 10 aM, (6) NC. **e** Gel electrophoresis of CASTSA products. (1) two-step CASTSA, (2) tsPCR, (3) non-Cas12a-treated control, (4) negative control. **f** Sequencing analysis of CASTSA amplicon, extracted from lane 1 in (**e**)

The assay was validated in clinical samples collected from individuals with Human Papillomavirus (HPV) detecting the high-risk HPV 16 through the L1 region, a conserved gene commonly targeted for detection.^18,25^ The system demonstrated high discrimination against other high-risk HPV subtypes, including HPV 18, 33, 45, and 52. Combining CASTSA with a laser-induced graphene (LIG)-based electrochemical sensing platform allowed us to minimize non-specific adsorption and improve analytical figures of merit. Overall, CASTSA achieved a limit of detection (LOD) of 18 copies/reaction, demonstrating the potential for high specificity for pathogen subtype differentiation, advancing nucleic acid diagnostics in an assay that is easier to implement when compared with standard clinical workflows.

## Results and Discussions

### CASTSA detection principle

The CASTSA scheme can be divided into two distinct steps, uniquely both compatible with a one-pot implementation: (1) *cis*-cleavage of the target by the Cas12a RNP, and (2) terminal-specific quantitative PCR amplification (tsPCR) (Fig. 1a). In the first step, the Cas12a RNP specifically recognizes and binds to the target sequence, guided by the crRNA. In the presence of the protospacer adjacent motif (PAM) (*TTTN* for LbaCas12a, where *N* = *A*, *C*, or *G*),^26,27^ the Cas12a enzyme cleaves both strands of the target at specific sites, generating a partial strand with a free 5’ terminal. The TSP primer then binds to this sub-target sequence through its specific terminal region, T2c, and extends to the T1s region, forming a self-folded hairpin structure. The incomplete hairpin structure subsequently extends the complementary region, Uc, at the 3’ end, completing the hairpin. The qPCR template is formed when the UP sequence, complementary to Uc, binds to the hairpin structure and extends the complementary regions Us and T3s at the 3’ and 5’ ends, respectively. To ensure accurate template formation, an iSpacer 18 blocker is inserted between regions U1s and T1s of the TSP. This blocker terminates the extension of UP at T1s on the hairpin structure, thereby generating a template containing qPCR primer-binding regions for UP and T3P at both termini. Although the addition of iSpacer18 does not completely prevent non-specific extension caused by mis-pairing between TSP primers in the T2s and T1s regions, the resulting double-stranded products do not contain probe regions, thus maintaining detection specificity. qPCR amplification is carried out using only the primers UP and T3c.

A critical feature of CASTSA is that amplification is only activated when both the CRISPR/Cas system and the TSP primer function as designed, ensuring specificity. The Cas12a system requires both the complementary crRNA region and the PAM sequence for successful target recognition and cleavage. If Cas12a does not cleave the target, even if TSP binds, extension will fail, and the hairpin structure cannot form, preventing amplification. This “Cas12a recognizing-to-PCR amplifying” process prevents nonspecific amplification, addressing issues found in earlier CRISPR-based diagnostic systems, where pre-amplification often led to false positives. The unique principle simplifies the operation and allows the one-tube reaction, providing a fully enclosed environment to prevent contamination. Furthermore, the inclusion of the UP sequence provides greater specificity compared to traditional double-specific-primer amplification systems. Collectively, these measures Cas12a recognition, TSP primer action, and the use of UP contribute significantly to the high specificity of CASTSA.

The CASTSA mechanism was validated in two stages: confirming Cas12a RNP cleavage and testing the feasibility of tsPCR initiated by TSP. The *cis-*cleavage site of the specific LbaCas12a RNP is located 18 bases downstream from the PAM sequence^26^ (Fig. 1b). A pair of primers positioned on either side of the cleavage site was designed to amplify the target sequence, except when Cas12a cleaves the double-strand target (Fig. 1b). Amplification of the HPV16 artificial template treated with Cas12a showed significantly lower efficiency compared to the untreated group, indicating that Cas12a effectively cleaved the target into partial strands (Fig. 1c). The fluorescence signal in the Cas12a-treated group was observed approximately three cycles later than in the untreated group, corresponding to a 10-fold decrease in the available intact template for amplification, suggesting a cleavage efficiency of ca. 90%.

To perform tsPCR independently, a single-strand oligonucleotide was synthesized spanning from region T1s to T2s on the target, simulating the cleavage product produced by Cas12a. This cleaved template was captured by TSP, formed into the functional hairpin structure, and amplified via tsPCR (Fig. 1d). By transferring the cleaved product of the intact double-stranded template into the tsPCR reaction mixture, the two-step CASTSA process was conducted. Only the Cas12a-treated template could be amplified by TSP-mediated PCR, producing a fluorescence signal. The amplification products were analyzed through electrophoresis and sequencing, confirming that the sequence matched the expected theoretical amplicon: 5’-Us-T1s-T0s-T3s-3’ (Fig. 1e, f).

### Optimization of CASTSA assay in one-tube

**Fig. 2:**
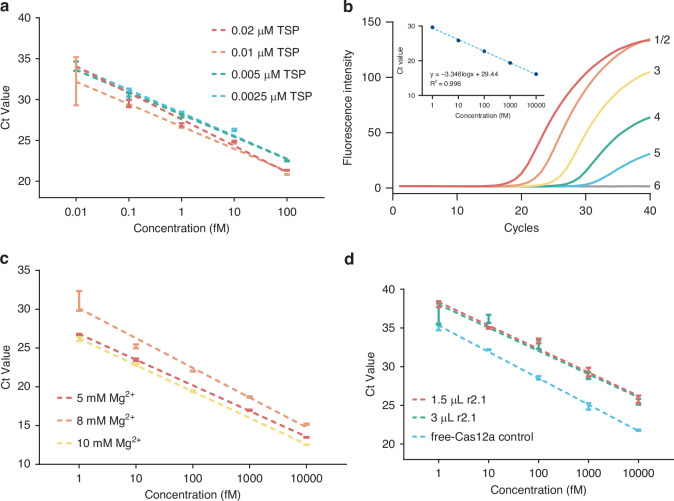
Optimization of one-tube CASTSA. **a** Optimization of TSP concentration for tsPCR amplification performance. **b** Feasibility of two-step CASTSA. (1) 10 pM, (2) 1 pM, (3) 100 fM, (4) 10 fM, (5) 1 fM, (6) NC. **c** Adjustment of magnesium ion concentration to fit one-tube CASTSA, showing no amplification under 2 mM Mg^2+^. **d** Comparison of cleavage efficiency between 5 mM Mg^2+^ (1.5 µl buffer r2.1) in one-tube CASTSA system and 10 mM Mg^2+^ recommended by NEB LbaCas12a instructions. Data are presented as mean ± SD (*n* = 3)

The amplification efficiency of the tsPCR step is highly sensitive to TSP concentration, as it links the Cas12a cleavage product to the qPCR template. Low TSP concentrations result in fewer hairpins and templates, reducing amplification efficiency, while high concentrations increase the risk of non-specific hybridization between the T1s and T2c regions, leading to spontaneous hairpin formation and non-specific amplification. As shown in Fig. 2a, 10 nM TSP was identified as the optimal concentration, yielding the highest efficiency. Under the optimal TSP concentration, the Ct value of two-step CASTSA was linearly related to the logarithm of the concentration of the template (Fig. 2b).

By integrating tsPCR and CRISPR-Cas cleavage in a single tube (or pot), CASTSA eliminates the contamination risks associated handling (e.g., lid opening) in earlier two-step PCR-based CRISPR-Dx systems. However, the buffer composition, particularly the magnesium ion (Mg^2+^) concentration, is often challenging for Cas12a and polymerase activities. The standard CRISPR cleavage reaction uses 10 mM Mg^2+^, while PCR typically requires 2 mM. Excess Mg^2+^ can cause non-specific amplification in PCR, whereas insufficient Mg^2+^ inhibits Cas12a activity. To optimize both reactions, Mg^2+^ concentration was tested from 2 mM to 10 mM. Figure 2c shows that 5 mM Mg^2+^ concentration provided the best balance, with no non-specific amplification in the blank group. This corresponds to half of the recommended quantity (3 µl) in NEB instruction of buffer r2.1 (1.5 µl). While 10 mM Mg^2+^ achieved the highest efficiency, it caused non-specific amplification in the negative control. At 2 mM, Cas12a was inactive, resulting in no amplification. Additionally, reducing the buffer volume from 3 µl to 1.5 µl maintained comparable *cis*-cleavage efficiency, confirming that Cas12a activity remained unaffected (Fig. 2d).

### Electrochemical platform

**Fig. 3:**
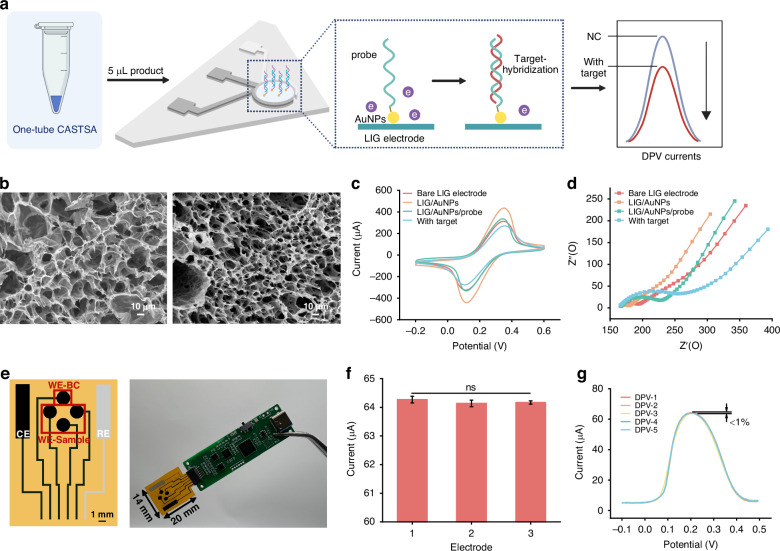
Graphene-based electrochemical device for one-tube CASTSA analysis. **a** Scheme of LIG electrochemical assay. **b** SEM images of bare LIG electrode (left) and AuNPs modified electrode (right). CV (**c**) and EIS (**d**) responses of the electrochemical chip in 0.1 M KCl, 5 mM K_3_FeC_6_N_6_, 5 mM K_4_[Fe(CN)_6_] solution after each modification: bare LIG electrode (red), AuNPs modified LIG electrode (orange), AuNPs/probe modified LIG electrode (green) and with positive one-tube CASTSA amplicons (blue). **e** Schematic diagram of the four-array electrochemical sensor. Three parallel working electrodes are used to analyse the sample and one electrode is used as a blank control (left). WE working electrode, BC blank control, CE counter electrode, RE Ag/AgCl reference electrode. The integration of the sensor into a portable electrochemical reader for electrochemical measurement and signal processing is illustrated in the right panel. Repeatability of the LIG/AuNPs electrochemical chip in three parallel electrodes (**f**) (*n* = 3) and in the same assay 5 times (**g**)

Whilst sensing of CRISPR-Dx signals by electrochemistry has been explored,^28^ our new approach using CATSA with an LIG-based electrochemical detection platform not only eliminates background interference from crude sample handling in fluorescence detection, thereby enhancing sensitivity, but also maintains the method’s inherent specificity. The electrochemical platform was constructed using a LIG electrode fabricated by CO_2_ laser irradiation on polyimide (PI) films^29^ (Fig. 3a). Formed by the disruption of chemical bonds in PI and recombination of carbon atoms at ultrahigh temperatures,^30,31^ the electrode exhibits high porosity (Fig. 3b), providing a high specific surface area for subsequent modification and immobilization of bio-elements. The results of the High Resolution Transmission Electron Microscope (HRTEM) confirmed the presence of graphene (Supplementary information Fig. S1). The Raman spectrum showed three characteristic bands of graphene-derived materials, including the D band at 1355 cm^−1^, the G band at 1585 cm^−1^ and the 2D band at 2701 cm^−1^ (Supplementary information Fig. S2), consistent with previously reported data.^32^ The ratio of the intensity of the peak for the D band with that of the G band was 0.366 indicating a high degree of graphitization and excellent conductivity. Parameter optimization was performed to maximize the electrical conductivity of the LIG-electrode. The measurement results of LIG-electrode resistance showed that its conductivity was highest when the processing speed was 60 mm/s, the light intensity was 15%, and the scanning accuracy was 200 dpi (Supplementary information Figure S3).

Gold nanoparticles (AuNPs) were modified through electro-deposition (Supplementary information Figure S4) to enhance the conductivity^33^ (Fig. 3c). HPV 16 probes were immobilized using a Au-S thiol bonding mechanism with AuNPs to capture CASTSA amplicons.^34^ Hybridization of the amplicons with the probes reduced surface conductivity, altering the current response. The changes in peak currents in cyclic voltammetry (Fig. 3c) confirm the successful modification of AuNPs. After modification with AuNPs, the peak current in cyclic voltametry (CV) measurements increased from 313 mA to 436 mA. Subsequent immobilization of capture probes reduced the peak current to 336 mA. Comparative analysis of pre- and post-hybridization detection curves revealed a significant current decrease (~20%) upon target DNA binding, showing the functionality of the modified electrode. These were further supported by the corresponding changes observed with electrochemical impedance spectroscopy (EIS) (Fig. 3d), consistent with prior work.^29^

To further address the critical issue of non-specific adsorption, an array device comprising a four electrochemical electrode configuration was used (Fig. 3e), with three electrodes modified with specific probes, while the fourth one remained unmodified, to be used to subtract background and noise. This approach minimized the influence of non-specific adsorption and improves the accuracy of the measurements. Paired with a portable, custom designed electrochemical potentiostat, the methods enable quantitative analysis in point-of-care testing (POCT). The electrochemical workstation circuit uses a compact, portable 58 × 18 mm four-layer PCB with a thickness of 1.6 mm. One end of the PCB is equipped with an FPC connector to accommodate sensors, and the other end features a USB-C interface that can connect to a battery or power supply. Its microcontroller is capable of simultaneously processing signals from four working electrodes without compromising the signal acquisition of the electrochemical sensors.^29^ The consistency and reproducibility of the parallel working electrodes were evaluated (Fig. 3f), with each electrode tested five times (Fig. 3g). Consistent differential pulse voltammetry (DPV) response curves were observed across all devices, with a variation of less than 1%, demonstrating the reliability of the platform.

### Detection performance of CASTSA platform

**Fig. 4:**
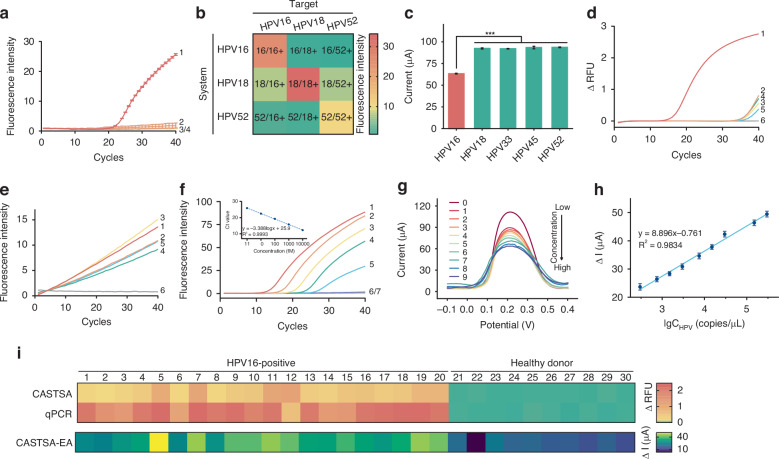
Analytical performance and clinical validation of one-tube CASTSA with electrochemical device. **a** Specificity of one-tube CASTSA by detecting (1) HPV 16, (2) HPV 18, (3) HPV 52, (4) NC. **b** Cross specificity of one-tube CASTSA using HPV 16, 18, and 52 detection systems. The fluorescence intensity of different systems results from different fluorescence moiety: HPV 16, 6’FAM; HPV 18, VIC; HPV 52, ROX. **c** Selectivity of electrochemical device by detecting one-tube CASTSA products of different-HPV subtype-infected clinical samples. Data is presented as the current change caused by the hybridization between HPV 16 probe, and the corresponding target as mean ± SD (*n* = 3), *** (*p* < 0.005). Specificity of qPCR (**d**) and *trans*-cleavage after pre-PCR in conventional CRISPR-Dx approach (**e**). (1) HPV 16, (2) HPV 18, (3) HPV 33, (4) HPV 45, (5) HPV 52, (6) NC. **f** Sensitivity of one-tube CASTSA for detecting HPV 16. (1) 10 pM, (2) 1 pM, (3) 100 fM, (4) 10 fM, (5) 1 fM, (6) 100 aM, (7) NC. **g** DPV performances of the electrochemical device against different concentration of targets after one-tube CASTSA detection. (0) blank, (1) 3*10^2^ copies/µl, (2) 0.75*10^3^ copies/µl, (3) 1.5*10^3^ copies/µl, (4) 3*10^3^ copies/µl, (5) 0.75*10^4^ copies/µl, (6) 1.5*10^4^ copies/µl, (7) 3*10^4^ copies/µl, (8) 1.5*10^5^ copies/µl, (9) 3*10^5^ copies/µl. **h** Calibration curve between the DPV currents and the logarithm of the concentration of HPV 16 template. Data are presented as mean ± SD (*n* = 3). **i** The results of clinical sample analysis respectively using one-tube CASTSA (top), real-time qPCR (middle), and one-tube CASTSA-electrochemical assay (bottom)

The specificity of the CASTSA system was validated using DNA genomes of various HPV subtypes extracted from clinical samples obtained from individuals with HPV. We demonstrated the specificity of the HPV 16-targeted CASTSA system by showing that the assay exclusively amplified HPV 16 DNA, with no detectable cross-reactivity to HPV 18 or HPV 52 under standardized testing conditions (Fig. 4a). To further assess cross-specificity, systems detecting HPV 18 and HPV 52 were also designed. The target regions for HPV 18 and HPV 52 were different from HPV 16, as the same region might lack a PAM sequence for CRISPR recognition. Additionally, it could lead to mis-binding of tsPCR-related primers, especially primer T2s and the associated probe. All primers across different systems were subjected to cross-binding validation to ensure specificity. The DNA genome of three subtypes was individually detected using three separate CASTSA assay systems. Positive results were observed only for their corresponding targets, confirming the high specificity of CASTSA system (Fig. 4b). The slightly higher background in the HPV 18 system may result from own characteristic of the dye VIC. The high specificity of CASTSA was further demonstrated by significant differences in electrode surface currents between HPV16 and other HPV subtypes (HPV 18, 33, 45, 52) (Fig. 4c).

In contrast, traditional qPCR targeting the same region exhibited non-specific signals across all four subtypes (Fig. 4d). Similarly, conventional CRISPR-Dx methods^35^ with pre-amplification showed reduced disparity in fluorescence intensity between HPV 16 and other subtypes after *trans*-cleavage (Fig. 4e), highlighting our ability of solving specificity issues.

To evaluate sensitivity, serial dilutions of an artificial HPV 16 template (10 pM to 100 aM) was prepared in healthy serum. A linear relationship between cycle threshold (Ct) values and log[concentration] was observed between 10 pM to 1 fM (Fig. 4f). Dilutions of low-concentration samples were analyzed on the electrochemical chip, demonstrating excellent analytical performance with detectable current changes even at 300 copies/µl (Fig. 4g). The electrochemical assay achieved a limit of detection (LOD) of 18 copies/reaction (Fig. 4h), surpassing the sensitivity of fluorescence-based analysis (LOD of 30 copies/reaction).^36^

### Clinical validation of CASTSA using clinical samples

To evaluate the clinical feasibility of CASTSA, 30 samples were collected, including 20 HPV 16-positive swab specimens and 10 negative healthy swab samples, all previously having been diagnosed using a nucleic acid hybrid capture-2 assay. After a simple pre-analytical step involving spin-down, the samples were analyzed in a one-tube CASTSA, followed by electrochemical detection. For comparison, TaqMan real-time quantitative PCR (qPCR), the most widely used method for HPV nucleic acid detection,^37^ was performed targeting the same region as CASTSA. The diagnostic results from one-tube CASTSA (both fluorescent and electrochemical) were fully consistent with those of qPCR (Fig. 4i), demonstrating CASTSA’s potential for high clinical specificity. All healthy samples showed low fluorescence signals, further confirming the reliability of the assay for clinical applications.

## Discussion

We developed CASTSA, a novel one-pot nucleic acid amplification method based on the CRISPR/Cas12a system and qPCR, designed to eliminate non-specific amplification inherent in the pre-amplification step of traditional CRISPR-Dx approaches. In CASTSA, amplification is initiated only after the Cas12a RNP specifically recognizes and cleaves the target, generating a free 5’ terminal. A key innovation is the design of a new TSP, which transforms the cleavage product into qPCR templates through self-folding and extension. Even if TSP binds to a non-target sequence with partial homology, it cannot extend or form a functional hairpin structure without *cis*-cleavage, ensuring high specificity by preventing non-specific amplification, an assay design which fully leverages the precision of the CRISPR system.

As a fully enclosed one-pot system, the protocol effectively prevents contamination, addressing a major limitation of traditional methods. Whilst CRISPR-Dx strategies have been implemented in a one-pot format, for example by combining with isothermal amplification^20^ or using the Cas protein alone,^38^ they usually use CRlSPR after amplification,^39^ contrary to our approach in CASTSA. Although CRISPR-direct digestion approaches have been explored, they rely on multi-site cleavages, necessitating multi-crRNA designs for a single target and stringent requirements on target region selection.^40^ To mitigate this, sgRNA fused by crRNA and tracrRNA has been used for CRISPR-Cas9 systems,^41^ but CASTSA offers a streamlined amplification solution through single-site cleavage on the target strand, significantly simplifying the reaction system, and also suitable for conventional CRISPR-analysis tools to initially choose crRNA recognition sites with better stability.

CASTSA demonstrated superior specificity in discriminating HPV16 from HPV18, 33, 45, and 52, while maintaining universal detection capability across multiple targets. The gold standard nucleic acid detection in clinical diagnosis remains traditional PCR. CASTSA effectively reduced the non-specific amplification of other subtypes when detecting a specific sequence, by incorporating CRISPR system upstream to directly recognize the original template in the sample. The Cas12a-crRNA complex enabled stringent identification with single-base specificity, even when discriminating highly similar sequence regions among pathogen subtypes. Although single-base specificity is possible in PCR platforms,^42^ it is only using stringent primer optimization and for limited organisms. To further compare the performance of the previous CRISPR-Dx for pathogen typing^43^ with CASTSA, the assay with pre-amplification followed by CRISPR was conducted to simulate the conventional concept of these studies. Results showed CRISPR cleavage failed to remove nonspecific amplicons from primer mis-binding during pre-amplification, confirming these methods’ specificity remains primer-dependent, a key limitation when targeting conserved viral subtype regions. These results further validated the significance of our reaction procedure of CRISPR-to-amplification and demonstrated the flexibility of CASTSA in target sequence selection compared to the previous methods. To enhance the specificity of pathogen typing at the point-of-care, isothermal amplification has often been integrated with additional platforms to mitigate the impact of nonspecific amplification such as digital microfluidics^44^ and spectroscopic analysis.^45^ CASTSA realized a one-pot CRISPR-Dx employing PCR instead of isothermal amplification, directly eliminating non-specific amplification and false positive.

A LIG-based electrochemical platform was constructed and utilized for sensitive end-point detection of amplification product. When combined with the electrochemical platform, CASTSA achieved a competitive LOD of 18 copies/reaction.^36^ The electrochemical biosensor with a unique four-array configuration, enhanced specificity and eliminated fluorescence crosstalk interference. Clinical validation using swab samples further confirmed its excellent analytical performance. When compared with the standard clinical nucleic acid detection workflows (including isothermal amplification kits), CASTSA demonstrated the potential for superior diagnostic specificity.

While CASTSA represents a significant advancement in specificity, its amplification performance showed slightly lower sensitivity compared to independent tsPCR. This may stem from the limited *cis*-cleavage efficiency of the CRISPR system, which rarely reaches 100%, as well as the suboptimal magnesium ion concentration required to balance both CRISPR and qPCR activities. Additionally, the potential for off-target effects in Cas12a recognition, although minimal, could also impact sensitivity.^46^ This is particularly relevant for digital nucleic acid detection, where the lower limit of target quantities for a single Cas-crRNA effector is a key parameter. Future work will focus on improving *cis-*cleavage efficiency and refining reaction conditions to further enhance sensitivity. However, these limitations are mitigated by the system’s high specificity and contamination-free design, which are critical for clinical applications.

CASTSA introduces a hairpin-mediated terminal-specific qPCR method using one UP and one TSP. To further enhance its utility, we are exploring the replacement of the TSP with a second UP sequence by refining the structure. This would establish a fully universal qPCR system for one-tube multiplex nucleic acid detection, maximizing CASTSA’s high-specificity amplification capability, and also expanding the versatility of CRISPR-based multiplexed assays beyond sole reliance on limited Cas protein variants^47^ or specialized high-throughput platforms.^48,49^ Such advances hold significant potential for precise subtype differentiation, mutation detection, and other applications requiring high specificity.

In conclusion, CASTSA addresses a critical challenge in nucleic acid detection, offering a robust, contamination-free platform with high specificity and sensitivity. Its integration with electrochemical biosensors and potential for multiplexing was used to demonstrate the potential for high specificity for pathogen subtype differentiation at the point of care, advancing nucleic acid diagnostics in an assay that is easier to implement when compared with standard clinical workflows.

## Materials and Methods

### Reagents and Instruments

All oligonucleotides were purchased from BiOligo Biotechnology (Shanghai) Co., Ltd. Champagne Taq DNA polymerase, PCR buffer, and dNTPs were obtained from Vazyme Biotech. EnGen LbaCas12a (Cpf1) and buffer r2.1 were purchased from New England Biolabs. The CRISPR incubation and the real-time amplification assays were performed on Roche 480. A total of 20 clinical vaginal discharge swap specimens positive for HPV16 infection, 5 positives for other subtypes including 18, 33, 45, and 52, all evaluated using standard clinical qPCR assays, and 10 healthy samples were collected between 2024 October 15 and October 23 by Adicon Clinical Laboratories, Hangzhou, 310023, Zhejiang, China. All samples characterization was done through standard nucleic acid hybrid capture-2 assay. This study was approved by the Ethics Committees of Adicon Clinical Laboratories (No. 2024-005), with all patients providing written informed consent. No compensation was provided to participants. The samples were prepared by brief centrifugation using a microcentrifuge (TGear) to collect the supernatant (~20 µl for each sample).

### Oligonucleotide primers and probes

The detailed sequence information of the HPV L1 gene was retrieved from the NCBI database. Two partially complementary primers, a forward and a reverse primer, were designed to amplify the target region via a linear PCR process with EvaGreen^TM^. The synthetic double-strand template covered the protospacer adjacent motif (PAM) to the TSP recognizing region (T2s). From the 5’ to 3’ end, the TSP contained a universal region (Us), a self-folding region (T1s), and a capture region (T2c). A Spacer 18 blocker was inserted between T1s and T2c, and partial bases near the 3’ end of T1s were modified with locked nucleic acids to enhance the affinity of accurate base pairing. The melting temperature (Tm) values, negative self-hairpin structure, and hetero-hybridization of all the primers were assessed using the IDT OligoAnalyzer tool. All the oligos’ information is shown in Table S1.

### Synthesis and quantification of double-strand templates

The 120 bp template was synthesized by two partially complementary paired primers PF_T and PR_T through a linear PCR. A 20 µl volume of mixture containing 0.2 µM PF_T, 0.2 µM PR_T, 1 × EvaGreen^TM^, 1 × PCR buffer (50 mM KCl, 1.5 mM MgCl_2_, and 10 mM Tris-HCl), 0.25 mM dNTPs, and 1 U of Taq DNA polymerase was reacted over 10 cycles at 95 °C for 10 s and 60 °C for 20 s. The product was purified, enriched by agarose gel electrophoresis and extracted using a HiPure Gel Pure DNA Mini Kit (Magen) according to the manufacturer’s instructions. The purified template was quantified using Nanodrop (Thermo Fisher Scientific).

### CRISPR-based qPCR assays

The CRISPR incubation was carried out in a 30 µl volume of reaction mixture containing 1 × buffer r2.1, 30 nM crRNA, 3 µl of template and 30 nM LbaCas12a, held at 37 °C for 30 min. In *cis-*cleavage validation, a 20 µl volume of reaction mixture containing 2 µl of CRISPR-treated product, 0.2 µM cutPF, 0.2 µM cutPR, 0.2 µM TaqMan probe, 1 × PCR buffer, 0.25 mM dNTPs, and 1 U of Taq DNA polymerase was reacted in 40 cycles at 95 °C for 10S and 60 °C for 20S. In a two-step CASTSA assay, a 20 µl volume of reaction mixture containing 2 µl of CRISPR-treated product, 0.01 µM TSP, 0.2 µM T3s, 0.2 µM UP, 0.2 µM TaqMan probe, 1 × PCR buffer, 0.25 mM dNTPs, and 1 U of Taq DNA polymerase was reacted over 7 cycles at 95 °C for 10 s and 60 °C for 90 s, followed by 40 cycles at 95 °C for 10 s and 65 °C for 20 s.

In the one-tube CASTSA assay, the CRISPR reaction components and TSP-mediated PCR reagents were combined to form a 30 µl reaction mixture. The 1× PCR buffer was replaced with 1.5 µl of 1× buffer r2.1, ensuring a final magnesium ion concentration of 5 mM for optimal reaction conditions. The reaction mixture was incubated at 37 °C for 30 min, followed by 7 cycles of the TSP extension at 95 °C for 10 s and 60 °C for 90 s, and then 40 cycles of PCR amplification at 95 °C for 10 s and 65 °C for 20 s.

### Amplicon analysis

The gel electrophoresis experiments were performed using 2.5% agarose gel in TAE buffer at 120 V for 45 min. The image was captured with an imager (GenoSens 2100, CLINX). The samples were sequenced by Sangon Biotech (Shanghai) Co. Ltd. The sequences were then checked using SnapGene Viewer (Dotmatics, version 4.1.9.0).

### LIG/AuNPs electrode fabrication and detection of amplicons

HPV 16 probe was synthesized by Sangon Biological Co. Ltd (Shanghai, China). Its sequence is shown in Table S1. Electrochemical equipment was purchased from Jiangsu Donghua Analytical Instrument Co., Ltd., China. LIG electrode preparation: polyimide (PI) films with a thickness of 100 µm were fixed on the substrate, using a CO_2_ laser cutter to fabricate a four-array LIG electrode in a preset pattern at 55 W, 100 mm/s, 5 mm laser spacing, a maximum light intensity of 20, and a minimum light intensity of 15. The reference electrode was then screen printed using conductive Ag/AgCl ink. After drying the chip, 5 µL of gold nanoparticles (AuNPs) purchased from MACKLIN were coated on the electrode surface and dried at room temperature for modification. 5 µM HPV 16 probe covered the electrode surface and were incubated at 45 °C for 30 min for immobilization. Then an excess of 5 µM MCH was added and incubated for 30 min to block the unmodified site on the substrate. Cyclic voltammetry (CV) was performed in a solution containing 0.1 M KCl, 5 mM K_3_[Fe(CN)_6_], and 5 mM K_3_[Fe(CN)_6_] over a potential range of −0.1 to 1.0 V at a scan rate of 100 mV/s for 10 cycles.

5 µl of the one-tube CASTSA amplicons were added to the electrochemical device and incubated for 30 min under room temperature. Then, the remaining amplicons were removed with pure water. After drying the chip, the electrochemical properties were characterized with differential pulse voltammetry (DPV) techniques in 0.1 M KCl, 5 mM K_3_FeC_6_N_6_, 5 mM K_4_[Fe(CN)_6_] solution. DPV was performed in the potential range of −0.1 to 0.5 V, with a step potential of 5 mV, a modulation time of 0.025 s, and an interval time of 0.5 s.^50^

## Supplementary information

Supplementary Information
