# Supplementary material for: One-pot CRISPR-based point of care platform for rapid, specific and sensitive detection of HPV 16 without pre-amplification
Source: Microsyst Nanoeng. 2026 Mar 9;12:81. doi: 10.1038/s41378-025-01130-y (PMC12972143; doi:10.1038/s41378-025-01130-y)
Supplement: Supplementary file 1 — Supplementary Information [file 41378_2025_1130_MOESM1_ESM.pdf]

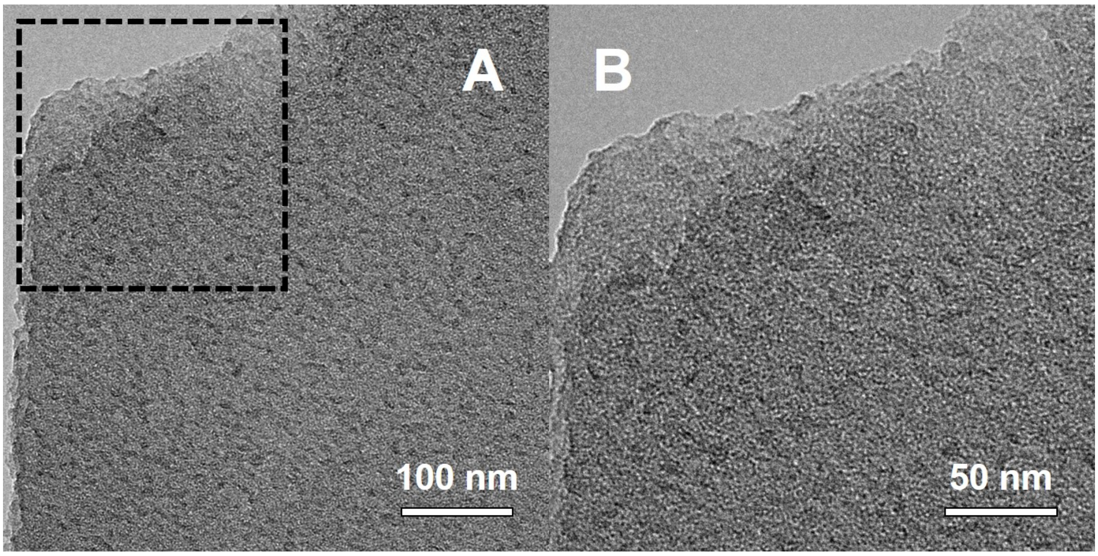

Fig. S1. HRTEM images of LIG-electrode. (A) scale bar 100 nm, zoomed in (B) following box plotted on (A).

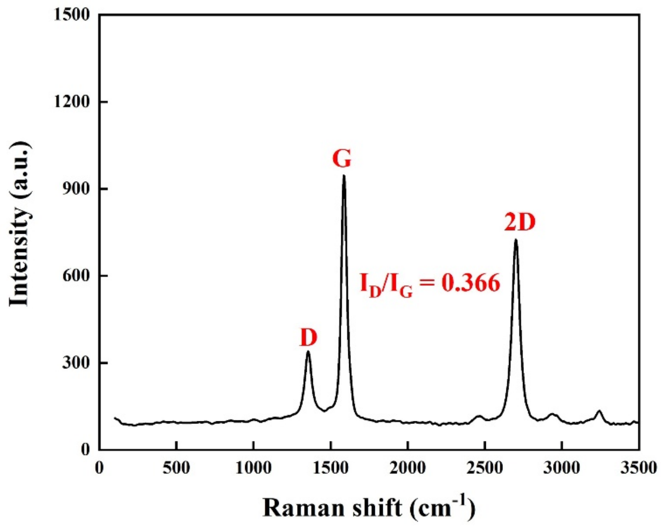

Fig. S2. Raman spectra of LIG-electrode.

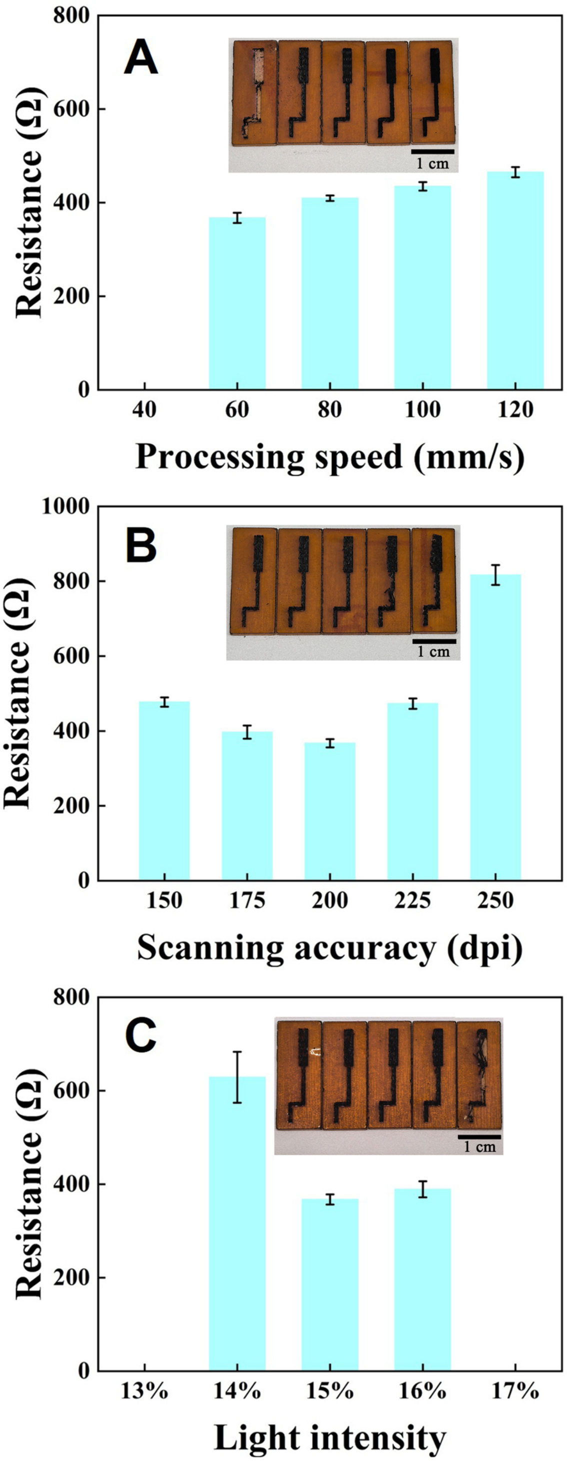

Fig. S3. The effect of processing speed (A), light intensity (B) and scanning accuracy (C) on LIG-electrodes resistance. Scale bar in inserts is 1 cm.

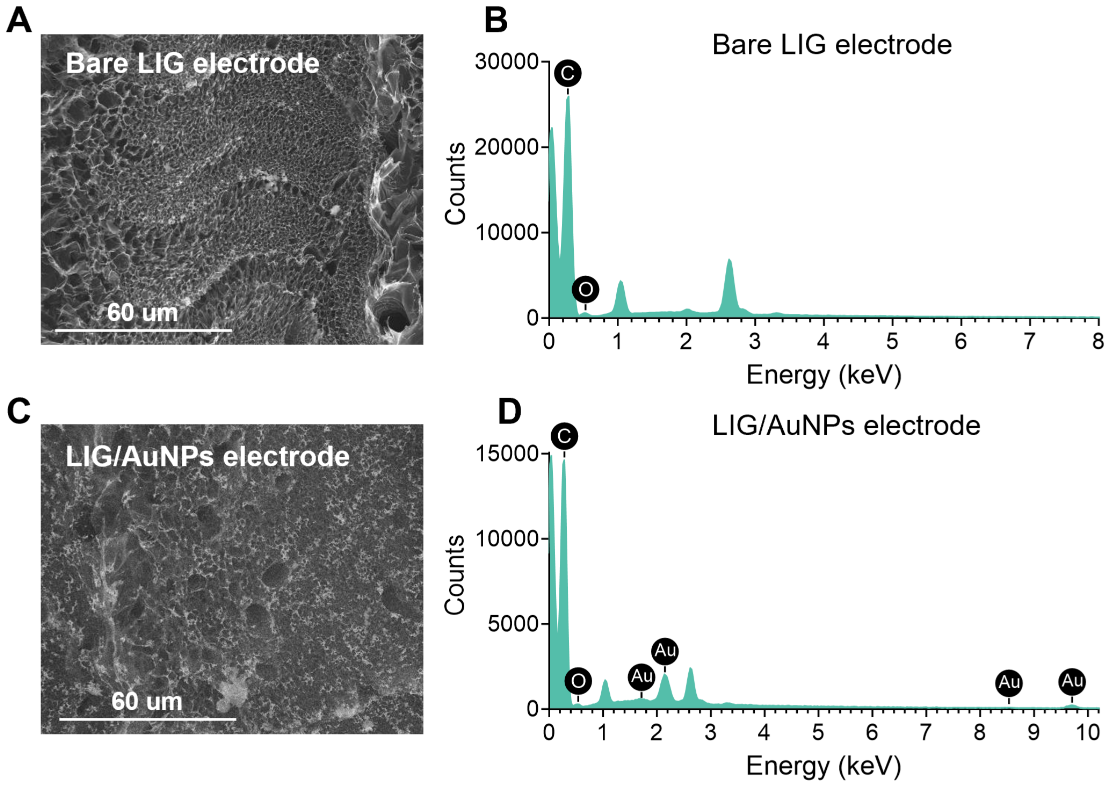

Fig. S4. EDS (Energy Dispersive Spectrometry) characterization of bare LIG electrode (A, B) and AuNPs modified electrode (C, D). The elements of the peaks were labeled.

Table. S1 Oligonucleotide primers and probes

Name	Sequence
PF-T	ACCTGCATTTGCTGCATAAGCACTAGCATTTTCTGTGTCAT CCAATTTATTTAATAAAGGATGGCCACTA
PR-T	GCCTGTGTAGGTGTTGAGGTAGGTCGTGGTCAGCCATTAG GTGTGGGCATTAGTGGCCATCCTTTATTAA
T1T0T2	T TTCTGTGTCATCCAATTTAT TTAATAAAGGATGGCCACTA ATGCCCACAC CTAATGGCTG ACCACGACCT ACCTCAACAC CTACACA
crRNA	UAAUUUCUACUAAGUGUAGAUCUGCAUAAGCACUAGCA UUU
TSP[1]	GGCTCACTCTCGCTCGCATCTTTCT+GTGT+CATC+CA+AT/i SP18/TGTGTAGGTGTTGAGGTAGG
T3P	GTGGTCAGCCATTAGGTGTG
UP	GGCTCACTCTCGCTCGCATC
probe	6’FAM-ATAAAGGATGGCCACTAATGC-MGB
cutPF (prePF)[2]	TTTGCTGCATAAGCACTAGC
cutPR (prePR)[2]	GTAGGTCGTGGTCAGCCATT
ssDNA reporter	6’FAM-TTATT-BHQ1
probe-EA[3]	ATAAAGGATGGCCACTAATGC-SH

[1]The ‘+’ in front of the base represents locked nucleic acid modification; ‘iSP18’ represents spacer 18 modification.

[2]The primer pair cutPF/cutPR for cleavage validation was also utilized for traditional quantitative PCR detection and pre-amplification in conventional CRISPR-Dx approach (prePF/prePR).

[3]The probe-EA was used in the electrochemical assay. ‘-SH’ represents sulfhydryl group modification.
